# Effect of Reducing Agent on Characteristics and Antibacterial Activity of Copper-Containing Particles in Textile Materials

**DOI:** 10.3390/ma15217623

**Published:** 2022-10-30

**Authors:** Remigijus Ivanauskas, Ingrida Ancutienė, Daiva Milašienė, Algimantas Ivanauskas, Asta Bronušienė

**Affiliations:** 1 Faculty of Chemical Technology, Department of Physical and Inorganic Chemistry, Kaunas University of Technology, 44249 Kaunas, Lithuania; 2 Faculty of Mechanical Engineering and Design, Department of Production Engineering, Kaunas University of Technology, 44249 Kaunas, Lithuania

**Keywords:** copper-containing particles, modification textile materials, antibacterial materials

## Abstract

Textile materials modified with copper-containing particles have antibacterial and antiviral properties that have prospects for use in healthcare. In the study, textile materials were saturated with copper-containing particles in their entire material volume by the absorption/diffusion method. The antibacterial properties of modified textile materials were confirmed by their inhibitory effect on *Staphylococcus aureus*, a Gram-positive bacterium that spreads predominantly through the respiratory tract. For the modification, ordinary textile materials of various origins and fiber structures were used. Technological conditions and compositions of modifying solutions were established, as well as the most suitable textile materials for modification. To assess the morphological and physical characteristics of copper-containing particles and the textile materials themselves, X-ray diffraction, a scanning electron microscope, and an energy-dispersive X-ray spectrum were used. In modified textile samples, XRD data showed the presence of crystalline phases of copper (Cu) and copper (I) oxide (Cu_2_O). On the grounds of the SEM/EDS analysis, the saturation of textile materials with copper-containing particles depends on the structure of the textile materials and the origins of the fibers included in their composition, as well as the modification conditions and the copper precursor.

## 1. Introduction

Due to the large surface-to-volume ratio and quantum effects, metal nanoparticles have amazing sensitivity to ultraviolet radiation, as well as thermal, electrical, catalytic, and antibacterial properties. These properties allow them to be widely used in sensor, optical, magnetic, and thermal devices, as well as in catalysis. Among metal nanoparticles, copper (Cu) nanoparticles are among the most widely used materials due to their lower cost, easy mixing with polymers [1], excellent electrical [2] and thermal conductivity [1,3], shorter reaction time compared with conventional catalysts [4], excellent solderability [5], low cytotoxicity [6], and antifungal and anticancer properties [7]. In the last decade, there has been a rapid increase in interest in textiles modified with copper particles, which acquire a number of useful properties, such as electromagnetic interference (EMI) shielding [8], high electrical conductivity [9], photocatalytic [10], hydrophobic [11], and especially antibacterial and antiviral properties [12,13,14]. It is known that copper and its nanoparticles have high reactivity and powerful antimicrobial [7,15,16] and antiviral activity [16,17,18]. Copper group metals silver and gold also have antibacterial [19] and antiviral [17] properties, but they are too expensive for application at the industrial scale. When bacteria and viruses are exposed to copper-containing substrates, their cell membrane is destroyed, which leads to the death of bacteria and viruses. Laboratory studies have shown that most types of bacteria die within a few hours on the surface of copper or its alloys [20,21]. With an increase in the concentration of copper in alloys, the efficiency and rate of the destruction of bacteria and viruses increase [18,22].

Since the SARS-CoV-2 virus, like influenza [23], is also an airborne virus, it can easily be transmitted through an abiotic surface contaminated with it [24,25]. Although the use of vaccines significantly reduces the risk of the spread of COVID-19, the continued spread of new strains of COVID-19 in the world has clearly demonstrated the poor preparedness of humankind to protect itself against airborne infections. At the beginning of July 2022, more than 546 million cases of infection and more than 6.3 million associated deaths were confirmed [26]. It is common knowledge that most people contract airborne infections through the respiratory tract, and the use of surface disinfectants offers little protection against infection. Therefore, considering the antiviral and antibacterial properties of copper, it is very promising to use filters saturated with copper-containing particles in the production of protective respiratory equipment. Depending on the composition, structure, and density of the filters, they would not only mechanically prevent bacteria and viruses from entering the human respiratory tract, but also effectively destroy them in the entire volume of the filter material due to contact with metal particles. It is likely that these materials could be used not only in the production of long-term personal protective equipment but also much more widely: for filtering indoor air or covering various surfaces in public places with which people usually come into contact.

Copper nanoparticles have been successfully obtained by physical and chemical methods such as laser ablation [27], vapor phase synthesis [28], high-pressure discharge [29], electrical discharges [30], microwave [31], chemical [32] and sonochemical reduction [33], electrochemical [34], hydrothermal [35], sol-gel synthesis [36], and microemulsion [37]. Unfortunately, most of these methods use complex equipment, high pressure or vacuum, making them extremely expensive. In addition, some methods require the use of hazardous reagents and toxic organic solvents and therefore are limited by environmental and biological risks and the vast majority of them are unsuitable for the introduction of copper-containing particles into textile materials. Among all the described methods, chemical wet processing is considered the simplest, most effective, and most suitable for the saturation of textile materials with copper-containing particles [38]. This process includes the reduction of copper cations in solutions of copper salts with a reducing agent to a metal with zero valency followed by blocking and stabilization of nanoparticles in textile materials of cellulosic origin [39,40,41] or non-woven polypropylene fabrics [42]. Thus, in view of the above, imparting antibacterial and antiviral properties to textile materials in a simple, environmentally friendly, easily controlled, fast, and energy-efficient method that can play a significant role in preventing the spread of airborne viruses, including SARS-CoV-2, in the current stressful situation caused by the pandemic.

The aim of the work was to select the most suitable ordinary textile materials of various compositions and structures without special preparation for saturation with copper-containing particles, as well as to select the most optimal conditions for modifying these materials. To achieve this, copper-containing particles were incorporated into the selected materials by the wet absorption/diffusion method, carrying out their synthesis in the entire volume of these materials. After this process, the next step was to establish that the modified textile materials acquired antibacterial properties, allowing further research on their use as filter components in the production of long-term protection.

## 2. Materials and Methods

### 2.1. Materials

The research used different types of textile materials with different fiber structures and compositions produced in four Lithuanian enterprises (Table 1). For the study, seven variants of non-woven materials produced by UAB “Neaustima” Šiauliai, Lithuania (hereinafter N1, N2, … N7), nine knitwear products by UAB “Omniteksas”, Kaunas, Lithuania (hereinafter O1, O2, … O9), eleven variants of AB “Utenos trikotažas”, Utena, Lithuania knitwear (hereinafter T1, T2, … T11), and two types of natural fiber fabrics produced by UAB “Klasikinė tekstilė”, Kaunas, Lithuania (hereinafter FH and F) were selected.

The following pure commercial reagents were used: from Sigma-Aldrich, (Taufkirchen, Germany) copper sulfate pentahydrate, CuSO_4_·5H_2_O (99.99% crystals and lumps), sodium bisulfite, NaHSO_3_ (≥99.0% crystals), and hydrazine hydrate NH_2_NH_2_· xH_2_O (50–60% reagent grade); from Merck (Darmstadt, Germany) sodium hypophosphite monohydrate, NaH_2_PO_2_·H_2_O (≥98.0% crystalline), ascorbic acid C_6_H_8_O_6_ (≥99.0% crystalline), and sodium borohydride (NaBH_4_, 98%, powder); and from Flucka (Oslo, Norway), L-ascorbic acid (C_6_H_8_O_6_, ≥99% powder).

### 2.2. Treatment Methods

**Figure 1:**
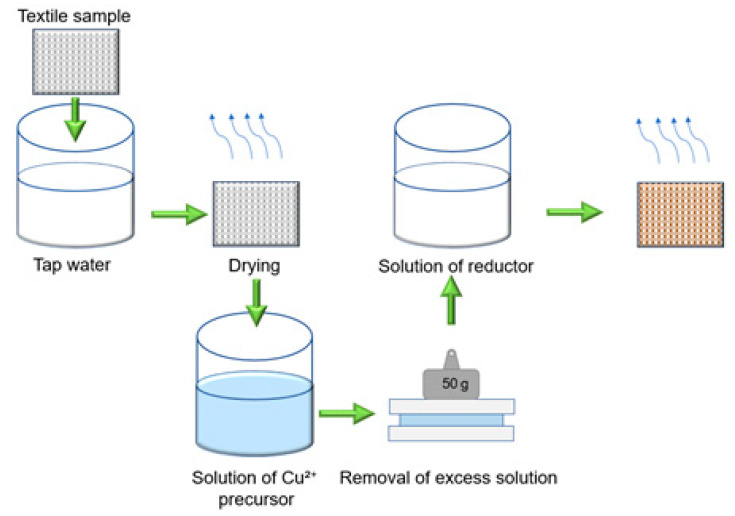
Scheme for the synthesis of copper particles in textile materials.

Antibacterial–antiviral layers of copper-containing particles in the entire volume of textile materials were formed directly by two-stage synthesis of these particles in the materials. Schematics of these procedures and the conditions for the formation of antibacterial-antiviral layers are presented in Figure 1 and Table 2. The compositions of the solutions used for modification were adapted from previous works [43,44,45,46,47,48], in which the synthesis of copper nanoparticles was carried out in solutions rather than in the entire volume of the textile materials, as in our case. Textile materials of 40 mm × 40 mm were used as substrates. Before processing, these samples were boiled in tap water for 30 min to improve their wettability so that they are more easily saturated with chemical reagent solutions. After that, they were dried and used in further research. At the first stage, samples of textile materials were impregnated with a solution of a copper precursor for 10 min at 25 °C, and then the excess solution was removed by clamping the impregnated samples between two glass plates. The relatively inexpensive and readily available copper sulfate pentahydrate was used as a source of copper precursor. At the second stage, Cu^2+^ cations in the entire volume of textile samples were reduced to elemental copper or its oxide. To complete this, textile samples saturated with copper sulfate and without its excess were exposed to solutions of various reducing agents (Table 2). After the second stage, the textile samples were dried for 12 h at 40 °C and used in further studies.

### 2.3. Investigative Methods

X-ray diffraction analysis of textile materials saturated with particles of copper or its oxide was performed using a D8 Advance diffractometer (Bruker AXS, Karlsruhe, Germany) operating at the tube voltage of 40 kV and tube current of 40 mA. Diffraction patterns were recorded in a Bragg–Brentano geometry using a fast-counting 1-dimensional detector Bruker LynxEye (Bruker AXS, Karlsruhe, Germany) based on silicon strip technology. The X-ray beam was filtered with an Ni 0.02 mm filter to suppress Cu-k alfa β-radiation and specimens were scanned over the range of 2*θ* = 5–60° at a scanning speed of 6° 1/min using a coupled two theta/theta scan type. The diffractometer is supplied with a software package called “DIFFRAC.SUITE” (Diffract.EVA.v.4.5, Bruker AXS, Karlsruhe, Germany). X-ray diffractograms of deposited layers were processed using the software package Search Match. 

Microphotographs (magnification ×56) of textile materials saturated with particles of copper and its oxide were obtained with an OLYMPUS SZX7 optical stereomicroscope (DF PLATO 1X_(-4), (Shinjuku-ku, Tokyo Japan).

SEM imaging was performed using the Scanning Electron Microscope (SEM) Quanta 200 FEG (FEI, Eindhoven, The Netherlands) operating in variable pressure mode, magnification–1000×, scale–100 μm, magnification–10,000×, 10 μm and magnification–2500×, 8 μm. Samples of textile materials saturated with particles of copper and its oxide were imaged under residual pressure of 80 Pa, which was sufficient to avoid imaging artefacts, e.g., sample charging, commonly resulting during the high-energy electron beam analysis. Energy dispersive spectroscopy (EDS) was performed using a Bruker XFlash 4030 detector (Bruker Corporation, Billerica, MA, USA).

The antibacterial effect of the modified textile materials was evaluated by their ability to inhibit the airborne Gram-positive bacteria *Staphylococcus aureus* ATCC 25923. For this, fresh 18 h cultures of bacteria were grown in peptone-soy broth (LAB-04, LAB-M) for 24 h at 37 °C. After culturing, the cells were mixed with a mini shaker and the turbidity of the suspensions was adjusted according to the McFarland No. 0.5 standard [49]. Then, the suspensions were introduced into a PCA Plate Count Agar medium cooled to 47 °C and 10 mL of the suspensions were pipetted into 90 mm diameter Petri plates. When the medium hardened, 8 mm diameter circles of the test textile material were placed on the surface and pressed. The plates were incubated overnight (18–24 h) at 37 °C. Circles of test textile material were used as negative control for bacteria at the corresponding growing conditions. Bacterial growth inhibition was determined by measuring the zone of inhibition (including the circles of test films diameter) appearing after the incubation period. Antimicrobial activity assays were repeated thrice in plate.

## 3. Results and Discussion

### 3.1. Selection of Solutions for Saturation of Textile Materials with Copper-Containing Particles

At the beginning, the selection of the most suitable textile materials for modification was carried out. To do this, samples of all textile materials (Table 1) were kept at 50 °C for 10 min in a solution of copper sulfate, then dried and tested. To complete this, it was observed whether these samples acquired an intense blue color, characteristic of the copper sulfate pentahydrate salt, which indicated the absorption of a large amount of Cu^2+^ ions by the textile material. Meanwhile, a slight shedding of crystals of this salt from these samples showed that copper sulfate crystals are strongly adsorbed on the surface of textile fibers and have good adhesion to their surface. Textile samples FH, N4, O1, O2, O6, T1, T2, T7, and T9 successfully met the above conditions.

In order to select solution compositions and experimental conditions for further research that would be universal and suitable for modifying textile materials of different structures and origins, we initially conducted tests with three textile materials of different structures. To complete this, samples of FH fabric, N4 nonwovens, and two samples of knitwear O1 and T1 were treated with solutions of all compositions (Table 2).

**Figure 2:**
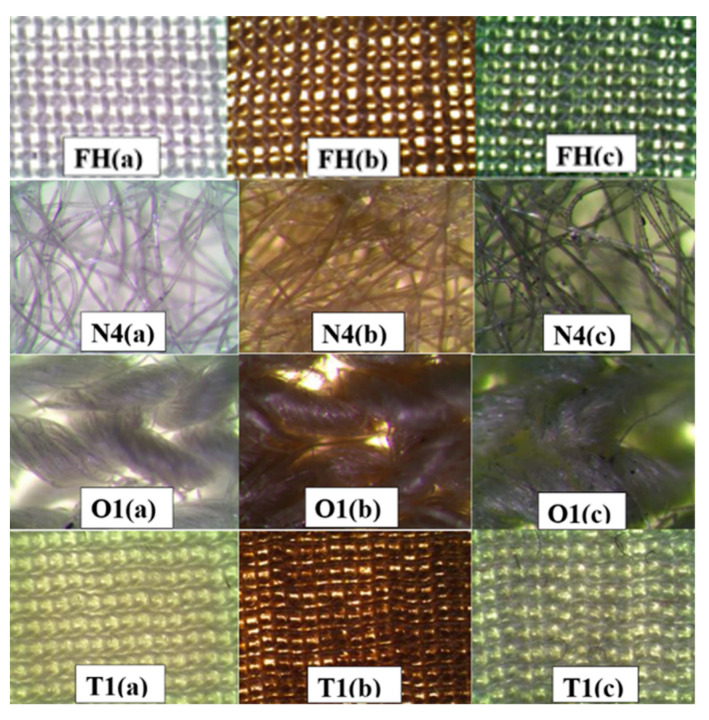
Micrographs of textile samples saturated with copper-containing particles. Raw material samples (**a**), material samples treated with reducer solutions No. 1 (**b**) and No. 2 (**c**).

When the samples of the material were immersed in the copper precursor solution, they acquired a light bluish color of the copper sulfate solution. After removing excess copper precursor solution from the material samples, they remained pale bluish in color. Subsequently, when the samples were placed in a reducing agent solution, they gradually changed their color from bluish to dark or light brown or even dark green (Figure 2). The color change of the samples indicated the completion of the reaction and the formation of copper-containing particles in the textile sample. The color intensity of the textile sample depended on the reducing agent used, as well as on the nature and structure of the modified material. When evaluating the samples of materials processed according to formulations No. 5 and No. 6, it was clearly seen that their surface was not covered evenly enough. In addition, cutting the materials showed that the copper-containing particles did not form in the entire volume. Moreover, copper particles easily fell out of the dried materials, which indicates that the formed particles do not have good adhesion to the surface of the fiber filaments, and that most of them are formed on the surface of the sample or closer to it. Better results were achieved with No. 5 only after increasing the number of modification cycles (copper precursor solution–reducing agent solution) to ten, while in other cases one cycle was sufficient. However, this increased the consumption of reagents and time, and, as a result, the cost of the process.

When using solution No. 6 to complete the reaction, a high temperature is necessary for the formation of copper-containing particles. Therefore, repeating this procedure partially changes the structure of natural fibers. As can be seen from the photographs shown in Figure 2, samples of textile materials treated with solutions of reducing agents No. 1–No. 4 were covered evenly. Only the colors of the samples differ, which may indicate a possible different composition of copper-containing particles. Therefore, X-ray diffraction analysis of the copper-containing particles in these samples was performed.

### 3.2. XRD Analysis of Copper-Containing Particles

X-ray diffraction is often used to analyze the structure of unknown crystalline materials and is a non-destructive testing technique. Therefore, it is very suitable in our work for the analysis of textile samples saturated with particles. The crystalline phases of the copper-containing particles were identified by comparing the measured pattern with archives in the XRD reference database. X-ray phase analysis of copper-containing particles was complicated not only by their large number of phases, but also by the high crystallinity of the fibers themselves [50,51,52,53,54], from which the textile materials used in the studies were made, as well as by the diversity of their origins. Therefore, X-ray diffraction patterns of only copper-containing particles shaken out of the modified textile samples were recorded. For a detailed interpretation of the X-ray diffraction data of the copper-containing particles, the data of the Joint Committee for Powder Diffraction Standards (JCPDS) and data available in the literature [55,56,57] were used.

**Figure 3:**
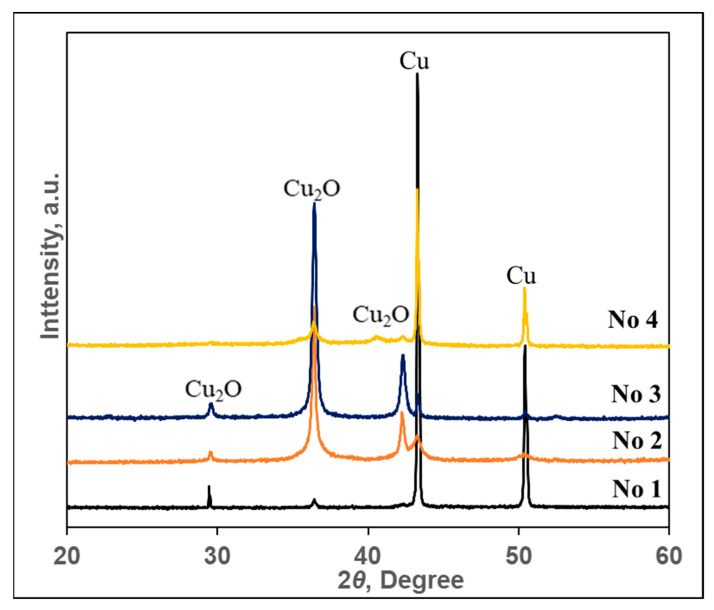
X-ray diffraction patterns of copper-containing particles obtained using solutions of various compositions. Peaks were identified and assigned as follows: cubic cuprite Cu_2_O (5–667) and cubic copper Cu (4–836).

X-ray diffraction results are shown in Figure 3 and the corresponding peak values are listed in Table 3. Part of Figure 3 shows X-ray patterns of particles obtained during the research, and part (b) shows X-ray patterns according to JCPDS data for comparison. The diffraction patterns were indexed to the two cubic phases of Cu and Cu_2_O, which are in good agreement with the reported data for Cu (copper, syn) (JCPDS Card File: 00-004-0836) and Cu_2_O (cuprite) (JCPDS Card File: 00-005-0667) (Table 3).

As can be seen from the diffraction pattern of the particles obtained using the No. 1 solution in part (a) shown in Figure 3, it is clearly dominated by only two peaks at 2ϴ 43.30 and 50.44°, referred to as cubic copper Cu (4–836). At that time, only two peaks of very low intensity cuprite Cu_2_O (5–667) were observed at 2ϴ 29.54 and 36.39°. Since the intensity of the peaks is simply proportional to the concentration, it can be stated that this layer consists of copper particles with traces of copper (I) oxide–cuprite. Particles very similar in composition, only with a slightly larger amount of cuprite, were obtained during their synthesis using the solution of composition No. 4. Meanwhile, in the particles obtained using solutions No. 2 and No. 3, cuprite dominated. As can be seen from the five peaks in the diffraction patterns of these particles, the two most intense ones at 2ϴ 36.39 and 42.25° and one with a lower intensity at 2ϴ 29.54° are attributed to cubic cuprite Cu_2_O (5–667). The remaining two peaks of low intensity at 2ϴ 43.30 and 50.44° belong to copper, which proves that the particles obtained using the No. 2 and No. 3 solutions contain a small amount of copper particles. In summary, the data of X-ray diffraction analysis show that particles obtained using the No. 1 and No. 4 solutions were dominated by copper, while cuprite dominated when the No. 2 and No. 3 solutions were used.

The data of X-ray phase analysis were indirectly confirmed by the change in the color of textile samples after their treatment with solutions of copper precursors. The layers dominated by copper particles became red, the color characteristic of elemental copper (Figure 2b). In addition, the layers dominated by cuprite particles acquired either a dark purple color, characteristic of copper (I) oxide, or a brown-green color, which is obtained by mixing cuprite paints and fabric samples (Figure 2c).

### 3.3. SEM and EDS Analysis of Textile Samples with Copper-Containing Particles

**Figure 4:**
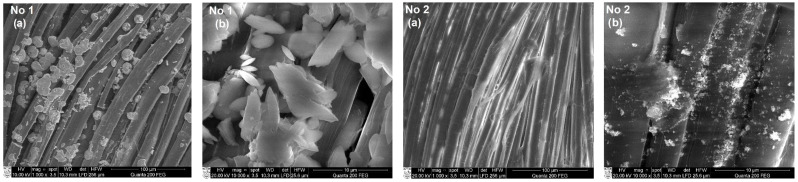
SEM images of fabric material sample FH (Flax/hemp, 50/50) treated with solutions No. 1 and No. 2. Magnification: (**a**) 1000×; (**b**) 10,000×.

**Figure 5:**
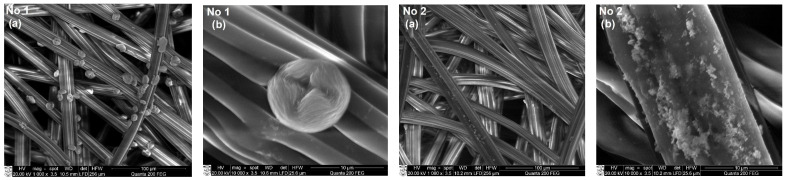
SEM images of non-woven material sample N4 (PES/viscose, 60/40) treated with solutions No. 1 and No. 2. Magnification: (**a**) 1000×; (**b**) 10,000×.

**Figure 6:**
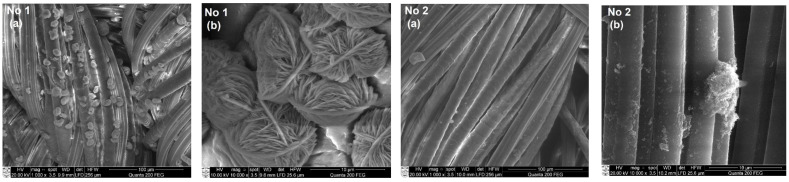
SEM images of knitted fabric materials sample O1 (wool/bamboo viscose, 60/40) treated with solutions No. 1 and No. 2. Magnification: (**a**) 1000×; (**b**) 10,000×.

**Figure 7:**
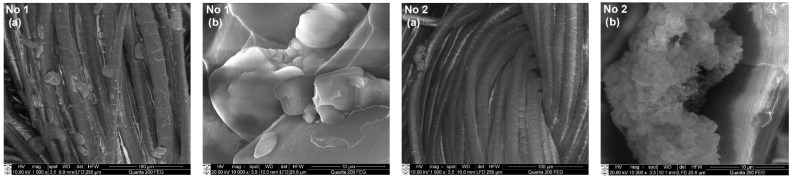
SEM images of knitted fabric material sample T1 (wool, 100) treated with solutions No. 1 and No. 2. Magnification: (**a**) 1000×; (**b**) 10,000×.

To assess the formation of copper-containing particles as a result of the adsorption/diffusion processes, the surface morphology and elemental composition of textile samples with these particles were studied by SEM and EDS methods. SEM images with a magnification of 1000× and 10,000× of various types of textile materials with different fiber structures and compositions (fabric (FH), non-woven fabric (N4), knitwear (O1, T1)) treated with solutions No. 1 and No. 2 are shown in Figure 4, Figure 5, Figure 6 and Figure 7. SEM images with different magnifications make it possible to observe not only the saturation of textile samples with particles containing copper but also the shape, morphology, and agglomeration of these particles. The SEM images show that various reducing agents such as ascorbic acid (C_6_H_8_O_6_) (solution No. 1) and sodium borohydride (NaBH_4_) (solution No. 2) also form particles of various shapes. As can be seen from the SEM micrographs of particles obtained with solution No. 1, the particles are very similar and have a spherical or hemispherical morphology, however, their agglomeration and overlay to textile samples of different origins are somewhat different. For example, particles on the surface of the fibers of textile samples O1 (wool/bamboo viscose, 60/40) and N4 (PES/viscose, 60/40) have an oval shape up to 10 µm in size (Figure 5 and Figure 6, No. 1 (a) and (b)). An almost continuous layer was formed on the fibers of a woolen textile sample (T1), on the surface of which individual oval-shaped particles or their agglomerates are visible (Figure 7, No. 1 (a) and (b)). Only the samples of flax/hemp fiber (FH) produced smaller particles ranging in size from 200 nm to 5 µm, some of which had an irregular shape (Figure 4, No. 1 (a) and (b)). The reason for this is probably the different origins of the textile samples, as well as differences in the processing of fibers in the production of these textile materials. Furthermore, as can be seen from the images at 1000×

magnification, the textile samples with the highest amount of natural fibers, especially wool (T1), are the most saturated with copper-containing particles. It is known that there is a relationship between the swelling of wool fibers in aqueous solutions and the pH value of these solutions. Qing et al. reported [58] that wool fibers swell least in the pH range of 5–7. Fiber swelling in aqueous solutions can be explained by a change in ionic interactions of charged acid-base groups of wool proteins with a change in pH. Thus, the good diffusion/absorption of Cu^2+^ ions into wool samples can be explained in our work by using a copper sulfate solution with pH 3.9. The greater adsorption capacity of Cu^2+^ cations by wool fibers in comparison with other cations was also noted by Monier et al. [59]. The swelling of the woolen textile material provided a high flux of dissolved Cu^2+^ cations penetrating into its depth and adsorbing them on the fiber surface. In addition, since the chemical composition of wool fibers is complex, with various functional groups, it is likely that the anionic forms of carboxyl groups (–COO^–^) included in its composition can form ionic bonds with adsorbed Cu^2+^. At the same time, free electron pairs of nitrogen from amino groups (–NH_2_) or oxygen from hydroxyl groups (–OH) can form donor–acceptor bonds with Cu^2+^. Finally, these cations were reduced to copper-containing particles in the second stage of the process. The influence of the pH of the reducing agent solutions confirmed the results, which were the best when using an ascorbic acid solution with pH 1.65 for the reduction of Cu^2+^ ions. Meanwhile, the pH of other reductant solutions was neutral or slightly alkaline. When NaBH_4_ was used to reduce Cu^2+^ ions (Figure 4, Figure 5, Figure 6 and Figure 7 (No. 2 (a) and (b)) it is clearly seen that the appearance of the resulting particles is completely different. On the surface of the fibers of textile samples, irregularly shaped particles up to 500 nm in size and smaller and their agglomerates are visible. The different shape and morphology of the particles can be explained by the use of ascorbic acid as a reducing agent, when particles of a different chemical composition were obtained with a predominance of cuprite Cu_2_O. The agglomeration of these particles and their overlay for textile samples of different origins, as well as particles obtained using the C_6_H_8_O_6_ reducing agent, are somewhat different. Moreover, as in the case of solution No. 1, samples of textiles of natural origin, especially from wool (T1), are saturated with these particles.

**Figure 8:**
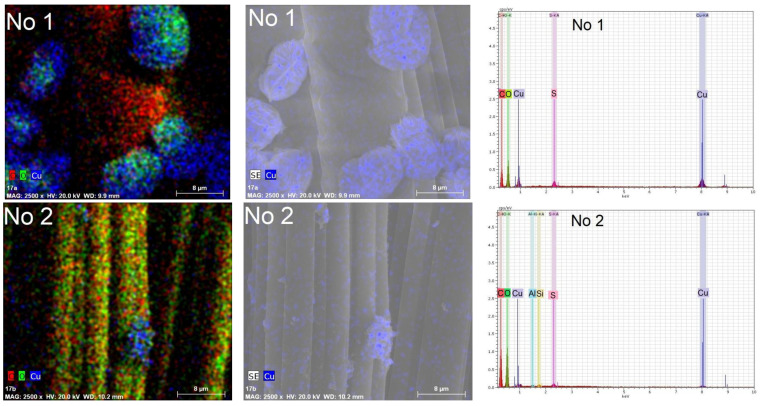
Chemical element maps and EDS spectra of knitted fabric materials sample O1 (wool/bamboo viscose, 60/40) treated with solutions No. 1 and No. 2. Magnification 2500×.

**Figure 9:**
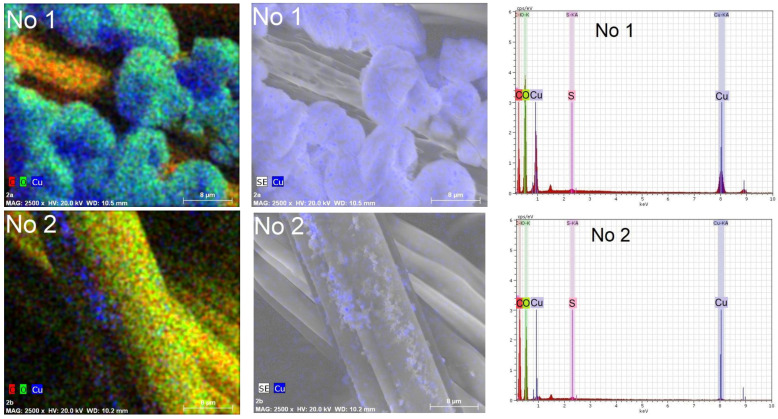
Chemical element maps and EDS spectra of knitted fabric materials sample N4 (PES/viscose, 60/40) treated with solutions No. 1 and No. 2. Magnification 2500×.

In order to confirm the presence of copper in the composition of the particles, elemental analysis by the EDS method was carried out on an area surface of 40 µm× 40 µm of the textile samples, as well as in their depth of several micrometers. Several observations can be made based on the element maps and EDS spectra presented in Figure 8 and Figure 9. First, copper predominates in the composition of all particles, regardless of the reducing agent used. In addition, it is embedded in the fibers of textile samples. This is especially noticeable in the sample of knitted materials O1 containing 60% wool. Secondly, the peaks at 0.56 eV, 0.94 eV, 8.04 eV, and 8.86 eV were associated with complex Cu X-ray lines. Moreover, their intensity is directly proportional to the content of copper in textile samples. Therefore, it can be concluded that there are more copper-containing particles in textile samples treated with solution No. 1. The remaining strong signals at 0.276 eV and 2.31 eV, respectively, are associated with the elements C and S, which are part of the textile fibers. In this case, the oxygen signal at 0.54 eV is associated with both the composition of textile fibers and the composition of cuprite Cu_2_O. The low-intensity peaks of Al at 1.48 eV and Si at 3.82 eV belong to the substrate on which the textile samples were placed during the EDS tests. The EDS results confirm the impression based on the analysis of visual SEM images that more copper-containing particles were formed in the textile samples when ascorbic acid was used to reduce Cu^2+^ ions.

### 3.4. Antibacterial Activity of Textil Materials with Copper-Containing Particles

For tests on bactericidal properties, the most common Gram-positive bacterium, *Staphylococcus aureus* (*S. aureus*, aureus staphylococcus), was chosen. It is spread by contact, like the COVID-19 virus.

As a rule, healthy carriers of staph or sick people can transmit staph to the surrounding airborne droplets, as well as through infected hands or the most diverse household items. Staphylococcus aureus is usually found in the nasopharynx or on the skin and can cause serious health problems ranging from mild skin lesions to life-threatening infections.

The antibacterial activity of all four textile samples was measured three times, and the results of this study are listed in Table 4. First, the bactericidal properties of all four untreated textile samples were studied. It showed that the untreated samples did not have a zone of inhibition, since their diameter was 0 mm. As can be seen from the data presented in Table 4, all textile samples treated with solutions No. 1 and No. 2 showed good inhibition of *S. aureus* bacteria, since the diameters of the zones of inhibition are ≥10 mm. The largest zone of inhibition (20–20–21 mm) and, consequently, the best antibacterial activity against *S. aureus* bacteria was shown by woolen samples T1. This is probably due to the ability of wool fibers to absorb more Cu^2+^ ions at the first stage of the process than fibers of other origins. As a result, the samples of woolen fabric were saturated with the largest amount of copper-containing particles after the end of the process.

It was stated that copper [18,60], its compounds, and copper-containing particles have antiviral properties in addition to antibacterial properties. Thus, according to the obtained test data for antibacterial activity, it can be expected that all textile samples containing Cu and Cu_2_O particles have an antibacterial effect. In recent years, a number of works have been published on the antiviral properties of copper-containing particles, including the inhibitory effect on SARS-CoV-2 [61,62,63,64]. Therefore, such modified textile materials have great potential for use in the manufacture of long-term protection products. However, this assumption needs to be confirmed in further studies.

Finally, it is important to note that, unlike other wet methods [39,40,41,42,65], saturation of textile materials with copper-containing particles by our method does not require high temperature, long duration, many technological steps, or additional special textile processing. By using this method, it is possible to modify the already prepared individual filter components of the mask, thereby simplifying and reducing the cost of their production.

## 4. Conclusions

Using the two-stage adsorption/diffusion method, textile samples of various compositions were saturated with copper-containing particles and their crystalline phase and morphological and chemical information were determined. Based on XRD data, the composition of copper-containing particles, including cubic copper (Cu, syn) (4–836) and cubic cuprite (Cu_2_O) (5–667), was determined, and which of these phases predominates in the composition of the particles depends on the reducing agent used. After the modification process, the color of the textile samples changed. Textile samples with a predominance of copper particles acquired a red color, characteristic of elemental copper, and samples with a predominance of cuprite particles acquired a dark purple color, characteristic of Cu_2_O. Based on the results of SEM analysis, it was found that the shape, size, degree of agglomeration, and the amount of copper-containing particles in textile samples depend on the reducing agent used, the structure of the textile samples, as well as the origins of the fibers from which they were made. The composition of the particles obtained using ascorbic acid as a reducing agent was prevailed by Cu (4–836), and their morphology were dominated by spherical or hemispherical particles with a size of 200 nm–10 µm, most of which are bound into agglomerates. When sodium borohydride was used as a reducing agent, Cu_2_O (5–667) predominated in the composition of the particles; the particles had an irregular shape up to 500 nm in size and less, and most of them were agglomerated. It was found that the most suitable substrate for saturation with copper-containing particles were textile materials, which were dominated by fibers of natural origin and especially wool. Meanwhile, as the amount of fibers of synthetic origin in textile materials increases, their deposition by copper-containing particles worsens. Moreover, for the reduction of adsorbed/diffused Cu^2+^ ions in textile materials to zero valency, L-ascorbic acid and sodium borohydride were best suited. It was found that the modified textile materials had good antibacterial properties and inhibited the airborne Gram-positive bacteria *Staphylococcus aureus*. Moreover, the diameter of inhibition zones of these bacteria, depending on the composition of the textile sample and the conditions of its modification, was within 10–22 cm. The combination of antibacterial and antiviral properties is very promising for mitigating the effects of the spread of disease-causing agents. Since the review of the literature showed that the particles synthesized in this work have not only antibacterial, but also antiviral properties, textile materials modified with these particles have great prospects for their use in the production of long-term protection.

## Figures and Tables

**Table 1:** Textile materials used in the research.

No.	Marking	Samples	Composition of Textile Materials, %	Weight, g/m^2^
1	FH	Fabric	Flax/hemp, 50/50	160 ± 8.0
2	F	Fabric	Flax, 100	160 ± 8.0
3	N1	Non-woven material (7K7–150)	Viscose, 100	150 ± 7.5
4	N2	Non-woven material (1K1A–130)	Polyester (PES), 100	130 ± 7.5
5	N3	Non-woven material (9A70W-050-03)	PES/viscose, 60/40	50 ± 2.5
6	N4	Non-woven material (9A70W-100-05)	PES/viscose, 60/40	100 ± 5.0
7	N5	Non-woven material (9A32W-090-03)	Polyester, 100	90 ± 4.5
8	N6	Non-woven material (1K1-220)	Polyester, 100	220 ± 11.0
9	N7	Non-woven material (9A70W-040-02)	PES/viscose, 60/40	40 ± 2.0
10	O1	Knitted fabric (7061S-1)	Wool/bamboo viscose, 60/40	150 ± 7.5
11	O2	Knitted fabric (7021 ENZ)	Cotton/hemp, 70/30	195 ± 9.75
12	O3	Knitted fabric (7222PM)	Modal viscose/milk protein fiber/lycra, 57/38/5	160 ± 8.0
13	O4	Knitted fabric	Cotton/bamboo viscose, 60/40	175 ± 8.75
14	O5	Knitted fabric	PES/hemp, 70/30	165 ± 8.25
15	O6	Knitted fabric	Tencel viscose/wool/lycra, 62/34/4	165 ± 8.25
16	O7	Knitted fabric	Recycled PES/hemp/PES/lycra, 57/25/16/2	270 ± 13.5
17	O8	Knitted fabric	Cotton/PES, 50/50	240 ± 12.0
18	O9	Knitted fabric	Bamboo viscose/cotton, 70/30	160 ± 8.0
19	T1	Knitted fabric (rib 1 + 1)	Wool, 100	180 ± 9.0
20	T2	Knitted fabric (Interlock), unpainted (finishing processes before painting)	Cotton, 100	200 ± 10.0
21	T3	Knitted fabric (single jersey)	Polyamide (PA), 100	160 ± 8.0
22	T4	Knitted fabric (double knit)	Cotton/PA, 88/12	370 ± 18.5
23	T5	Knitted fabric (rib 1 + 1), unpainted (finishing processes before painting)	Wool/acrylic Dralon fibers, NM 50/1, 50/50	200 ± 10.0
24	T6	Knitted fabric (single jersey), unpainted (finishing processes before painting)	Bamboo, 100	160 ± 8.0
25	T7	Knitted fabric (Interlock)	Wool/PA, 80/20	170 ± 8.5
26	T8	Knitted fabric	Cotton/PES, 84/16	330 ± 16.5
27	T9	Knitted fabric (Interlock), unpainted (finishing processes before painting	Cotton, 100	200 ±10.0
28	T10	Knitted fabric (single jersey)	Tencel viscose, 100	190 ± 9.5
29	T11	Knitted fabric (variegated rib)	Cotton/PES, 85/15	340 ± 17.0

**Table 2:** Conditions for saturation of textile materials with copper-containing particles.

No	Concentration of CuSO_4_·5H_2_O, mol/L	Reductant and Its Concentration, mol/L	Conditions for the Second Stage	
			Temperature, °C	Duration, minutes
1	0.5	C_6_H_8_O_6_, 0.6	60	60
2	0.05	NaBH_4_, 0.15	25	3
3	0.1	N_2_H_4_·H_2_O, 0.15	25	30
4	0.01	NaH_2_PO_2_·H_2_O, 0.02	80	120
5	0.125	NaHSO_3_, 0.125	60	10
6	0.1	C_6_H_12_O_6_, 0.2	100	20

**Table 3:** Comparison of experimentally determined inter-plane distances with JCPDS data.

2θ (Degree)	Phase	Inter-Plane Distances (d), Å	
		Experimental Data	JCPDS Data
29.54	Cu_2_O	3.029	3.033
36.39	Cu_2_O	2.466	2.465
42.28	Cu_2_O	2.136	2.135
43.30	Cu	2.088	2.088
50.44	Cu	1.808	1.808

**Table 4:** Antibacterial activity of textile samples with copper-containing particles.

Sample	Inhibition Zone, mm	
	Reducer Solution No. 1	Reducer Solution No. 2
FH	13–16–15	16–14–14
N4	12–12–13	15–16–14
O1	10–10–10	14–14–14
T1	20–22–21	18–18–19

## Data Availability

Not applicable.
